# The Double Challenges of Single Parents Raising Children with Disabilities

**DOI:** 10.1007/s10680-026-09767-9

**Published:** 2026-02-17

**Authors:** Nicoletta Balbo, Roxana-Diana Burciu

**Affiliations:** 1 Dondena Centre and Department of Social and Political Science, Bocconi University, Milan, Italy; 2 Centre d’Estudis Demogràfics, Barcelona, Spain

**Keywords:** Disability, Family, Well-being, Intersectionality, Single parenthood

## Abstract

This paper sheds light on the health-related, economic, and social well-being of single parents of children with disabilities, a population often overlooked in research. Much literature examines single parenthood and child disability independently and generally finds negative consequences of experiencing either. Yet, little is known about the well-being of parents at the intersections of single parenthood and raising a child with a disability. Drawing on data from the UK Millennium Cohort Study, the study compares the health-related, economic, and social well-being of single parents of children with disabilities to that of single parents of non-disabled children and partnered parents. The findings show that single parents of children with disabilities experience significantly worse mental and physical health, higher rates of poverty and lower employment rates. These challenges are compounded by the lack of appropriate childcare and limited support from non-residential parents and extended family members, which further constrains parents’ ability to work. This study investigates a neglected social group and contributes to the literature by integrating two separate strands of research—single parenthood and child disability—and identifying the unique disadvantages that stand at their intersection.

**Supplementary Information:**

The online version contains supplementary material available at 10.1007/s10680-026-09767-9.

##  Introduction

According to existing literature, child disability impacts the life of about 7 to 13% of children in the United Kingdom (UK) (Blackburn et al., 2010; Emerson et al., 2010). Yet, the impact of child disability is much more widespread, if we consider its effects on family members. As much as one in five families are affected by child disability, making it not just a personal challenge, but a population-level concern. Similarly, some scholars find that parents’ union dissolution is more common in families with a child with a disability compared to families with no disability (Urbano & Hodapp, 2007).^1^ Nevertheless, little is known about the mental health, care responsibilities, and economic well-being of parents of children with a disability after their transition to single parenthood. This paper asks *how raising a child with a disability impacts the well-being of single parents*.^2^ By examining the double-challenges of single parents of children with a disability, we aim to reveal the vulnerabilities and unique challenges faced by a group that is largely invisible to public policy and research.

In contrast to previous literature, we investigate a variety of well-being outcomes rather than focus only on labour market outcomes of parents with children with disabilities (Brown & Clark, 2017; Vinck & Brekke, 2020; Wondemu et al., 2022). Additionally, we also compare the well-being of single parents of children with disabilities with two other traditional and well-studied vulnerable groups: single parents of children with no disabilities and coupled parents of children with disabilities. To benchmark the results, we offer estimations of well-being in two-parent families with children with no disabilities. This builds upon previous studies but moves away from a two-way comparison of parents with or without children with disabilities (Wendelborg & Tøssebro, 2016) or from studies that exclusively investigate parents of children with disabilities (Cantero-Garlito et al., 2020).

Although much has been studied about single parenthood and child disability independently, what happens when these two challenges intersect is less clear. Single parents are disadvantaged compared to coupled parents in two main ways with implications for our study. First, they are often the sole caregivers of their children, which increases the conflict between caregiving and work, leading to unstable attachment to the labour market (Nieuwenhuis & Maldonado, 2018). Second, single parents in the UK are more often exposed to poverty and economic stain compared to coupled parents (Burciu, 2025). We argue that single parents of children with disabilities experience double challenges in caregiving due to their nature as sole caregivers and caregivers for children with enhanced care needs. This paper is one of the first research endeavours that argues that raising a child with a disability acts as a further, separate source of disadvantage for single parents, a population already exposed to high poverty risks (McLanahan, 2009). By exploring the intersectionality of singlehood and disability using a population-level analysis, we identify and describe the precarious – but often ignored – unique challenges that many single parents of children with disability experience.

Using longitudinal data from the UK Millennium Cohort Study (University College of London, 2024; 10.5255/UKDA-Series-2000031), which follows a cohort of children born in the UK in 2000–2001, this study provides population-level descriptive insights into the well-being of single parents raising children with disabilities. We show that single parents of children with disability report worse mental health and lower levels of physical health than single parents of children with no disability, partnered parents of children with disabilities, and partnered parents of children without disabilities. We also show that compared to partnered parents, single parents, and especially single parents of children with disability are more often in poverty, less frequently employed, receive less help from the non-residential parent and have less contact with their own parents. We build upon previous studies that show differences in employment among parents of disabled and non-disabled children (Vinck & Brekke, 2020) and explore several mechanisms that could explain these differences in labour market attachments. Our results show that many single parents of children with disability cannot work due to their increased care duties coupled with the lack of appropriate and affordable childcare. To allow single parents of children with disabilities to be employed, work arrangements that allow for flexible schedules, help with childcare, and generally promote family-work balance should be made available.

This study contributes to the existing literature by bridging and integrating two different strands of demographic, sociological, and public health research that have been mainly investigated independently: studies on single parenthood and those on child disability and family spillover effects. Child disability is associated with a higher risk of parental separation and divorce (Loft, 2011), therefore single parenthood and child disability are two conditions of disadvantage that can likely occur jointly. It is thus crucial to investigate how these two sources of inequality affect different dimensions of well-being for these families. Our results expose important lessons for research and policy by shedding new light on this double-disadvantaged social group and uncovering their challenges and needs. By comparing these families with both two-parent families having children with disability and single-parent families with healthy children, we can furthermore understand to what extent their challenges are similar to those of other social groups or whether they face a group-specific, uniquely disadvantaged situation.

##  Background

###  Child Disability and Its Reach

The definition of disability is highly debated in the literature (Blackburn et al., 2010), and the multi-dimensional and dynamic nature of disability complicates the attempts to formulate clear definitions even further. In the last 40 years, we have slowly moved from a medical model of disability, defined by the mere presence of an impairment, that can be physical, intellectual, or sensory, toward a bio-psychosocial model (Oliver, 1983). In line with this latter paradigm, the World Health Organization (2001) has defined disability as the result of the interaction between medical impairments and the barriers faced within the social environment which in turn leads to limitations in activities.

During these decades, in the attempt of translating the bio-psychosocial model into an effective measure of disability, scholars have proposed and challenged a broad range of measures of disability. Some studies use a criterion that includes a time requirement – a disability is only characterized as such if it is “longstanding”, usually more than 6 or 12 months (Blackburn et al., 2010; Emerson et al., 2010). Others identify children with disability as those who qualify to receive disability benefits (Brekke & Nadim, 2017; Vinck, 2022), ultimately relying on the guidelines set by policy, or who have special educational needs (Emerson et al., 2010). Some studies use specific limitations, for example, difficulties in the ability to learn (Powers, 2001), or specific diagnosis, such as Autism (Boyd, 2002) or Down syndrome (Urbano & Hodapp, 2007).

Bearing in mind these differences in defining and measuring child disability, in the United Kingdom (UK) between 7 and 10% of children have some form of disability (Blackburn et al., 2010; Emerson et al., 2010). Of these, a third of children experience mild limitations and 13.3% have severe limitations in their daily lives (Blackburn et al., 2010). This means that between 17 and 23% of British families have a child with a disability^3^ (Blackburn et al., 2010), representing a substantial proportion of the population that deals with the consequences of disability in their everyday life.

In this paper, we rely on a limitation concept of disability which classifies children with disabilities as those children whose parents report the presence of a condition that limits the ability of the children to undertake everyday activities, likely in a permanent way. Whereas the consequences of disability for the children, who themselves experience it, have been more extensively investigated (Chatzitheochari et al., 2016; Erickson & Macmillan, 2018), we argue that the impact on the families of children with disability is an important, but still overlooked, area of study, not only because of the large number of people who are affected by having a child with a disability in their family, but also because families, and specifically parents, are the main caregivers for individuals with disabilities. Understanding the consequences of child disability on families can allow researchers to better identify areas of need and potential sources of support for families with children with disability, promoting inclusion and successful family adaptation. In this paper, we consider the effects of having a child with disabilities on parents, specifically single parents.

### Consequences of Child Disability on Family Life

Why would a child’s disability affect family life, and parents in particular? According to the linked lives perspective, individuals do not live in isolation, but rather their lives are highly interconnected with those of other people (Elder et al., 2003). This interdependence is even stronger in close social cycles and in proximate social environments, such as families (Gilligan et al., 2018). As a result, family lives are multidimensionally linked, shaping outcomes among family members. Similarly, family system theory argues that children and parents reciprocally influence each other because no member of a family exists in isolation, they are all embedded in the family unit (Seligman & Darling, 2017). Children’s development is shaped by parents’ behaviour and parenting practices, which in turn affect parents (Hastings, 2002; Rigles, 2019). Therefore, the specific condition of each member affects the others, not only via a downward transmission – from parents to children – but also via an upward transmission – from children to parents, as well as horizontally – from sibling to sibling. This creates a multiplier effect.

Children with disabilities require more time and energy investment from their parents due to their heightened care needs (Powers, 2001; Vinck, 2022). This increased care responsibility is shown to affect three main areas of parents’ lives: economic participation, physical and mental health, and relationship quality.

Most of the attention of scholars has been concentrated on the labour market participation of parents of children with disabilities. Family members of children with disabilities have their participation in labour market impaired (Vinck & Van Lancker, 2020). Taking care of a child with a disability is usually a responsibility mainly shouldered by the mother, who therefore needs to juggle multiple roles, for example, as caregiver and employee (Brekke & Nadim, 2017; Schormans & Brown, 2004). For this reason, most of the time, mothers exit the labour market or reduce their working hours to accommodate the increased care needs of their children with disabilities (Jenkins, 1991; Powers, 2001; Reichman et al., 2008). The incentive to do so is even higher for women whose children are severely impaired, and for mothers with pre-school aged children (Brekke & Nadim, 2017; Vinck & Van Lancker, 2020). Mothers of children who have physical disabilities or limitations in their ability to take care of themselves show even lower labour supply compared to mothers with children who have mental, emotional, or sensory limitations (Wasi et al., 2011). Even in countries where the labour market participation of mothers with children having a disability is not different from that of mothers of healthy children, like Norway – characterized by a rather equal gender system—mothers of children with disability have lower earnings and longer sickness absence from jobs even (compared to mothers of children with no disabilities) four years after the birth of their children (Brekke & Nadim, 2017). Although many previous studies documented differences in labour market attachment between parents of children with and without disabilities (Brown & Clark, 2017; Vinck & Brekke, 2020), it remains unclear what are the mechanisms behind these differences.

A combination of preferences, social norms, structural barriers, and institutional factors could explain these differences. In this paper, we make use of detailed survey data to explore a variety of mechanisms that could explain why (single) parents of children with disabilities work less often than parents of children with no disabilities. An increasing number of studies investigate poverty outcomes among families of children with disabilities. Most children with disabilities live in low-income families (Blackburn et al., 2010). As a result, these children are substantially more likely to grow up in poverty compared to their peers (Fujiura & Yamaki, 2000; Powers, 2001). Families supporting a child with a disability are more likely to enter poverty and less likely to escape poverty compared to families who do not have a child with a disability (Emerson et al., 2010). Additionally, having a child with a disability implies additional costs related to medical treatments, social care, and special education that are uncommon among families with children with no disabilities (Jenkins, 1991; Reichman et al., 2008; Vinck, 2022; Vinck & Van Lancker, 2020; Wasi et al., 2011), and that can put further strains on the economic conditions of these families. Another factor that contributes to high poverty of families with children with a disability is their non-take-up of disability benefits (Vinck, 2022).

Beyond economic well-being, having a child with a disability is linked to increased parental stress (Olsson & Hwang, 2001; Reichman et al., 2008). Some studies investigate parental stress in families with children having a disability and argue that behavioural problems, rather than disability, explain increased levels of stress (Floyd & Gallagher, 1997; Hastings, 2002). Others argue that is not disability that is inherently burdensome, showing instead that it is ableism and poverty that lead to worse maternal health in families of children with disability, especially those with fewer socioeconomic resources (Bixby, 2023). Regardless of the cause, parents of children with disabilities report higher scores in terms of anxiety and depression compared to parents of healthy children (Hastings, 2002). Mental health issues of parents of children with disability are often positively associated with a lack of social support (Boyd, 2002).

Having a child with a disability influences the relationship quality between the parents, impacting living arrangements and family structure (Reichman et al., 2008; Risdal & Singer, 2004). Most scholars agree that parents of children with disability are less likely to get married and more likely to divorce (if they married at all) compared to parents of healthy children (Cohen & Petrescu‐Prahova, 2006; Hogan, 2012; Kim et al., 2023).

Although some studies find no effect of specific child disabilities on divorce (Anchesi et al., 2023; Müller et al., 2022), many studies highlight the increased union dissolution of families with children with disabilities (Loft, 2011), and the consequent higher risk for children with disabilities to live in single parent families. Yet little is known about the well-being of single parents with children having a disability, especially in comparison with single parents of children with no disability. This study proposes a comparison of single parents’ well-being between those with and without children with disabilities. The existing literature compares single parents of children with disabilities with two-parent families of children with disabilities (Grant & Whittell, 2000; Olsson & Hwang, 2001), but we believe that in order to shed further light on this potentially double-challenged group, a further and novel comparison with single-parents having healthy children is very important, as these two groups share common challenges, and characteristics that are specific only to single-parent families.

Extended family networks are an important resource for parents raising children with disabilities that can reduce their stress, their economic strain, and their time constrains (Novak-Pavlic et al., 2022). Grandparents, in particular, offer financial, emotional, and instrumental support to parents raising children with disabilities (D’Astous et al., 2013; Moffatt et al., 2019; Schilmoeller & Baranowski, 1998), by providing childcare, transportation, and economic help. Mothers of children with disabilities report that grandparents are an important source of support (Lee & Gardner, 2010) and the relationship between parents and grandparents is improved when grandparental involvement in childrearing is high (Mirfin-Veitch et al., 1997). Residential proximity and frequency of contact seem to be the best predictors (and thereby measures) of grandparental support to families of children with disability (D’Astous et al., 2013; Gardner et al., 1994). As a result, social support can moderate the relationship between parental well-being, family structure and child disability.

### Disability and Single Parenthood

Single parenthood is consistently associated with reduced well-being due to reduced employment opportunities (Biegert et al., 2022), high poverty rates (Edin & Kissane, 2010; Rank, 2000), and increased psychological strain (Harkness, 2016). Previous studies have not directly compared single parents of children without disabilities to partnered parents of children with disabilities. Consequently, it is unclear whether the presence of a partner or the child’s disability status plays a more central role in shaping parental well-being. Given robust evidence that partnership provides social, emotional, and economic support (Cutrona, 1996), we expect that single parents will exhibit lower well-being than partnered parents regardless of whether their children have a disability.

Some studies have found that children with disabilities live in single-parent households, more often than non-disabled children (Blackburn et al., 2010; Cohen & Petrescu‐Prahova, 2006; Fujiura & Yamaki, 2000; Hogan, 2012; Vinck, 2022). The two main reasons behind this trend are the increased divorce rates among parents of children with disability (Hogan, 2012; Kim et al., 2023), as well as the lower likelihood of getting married in the first place (Cohen & Petrescu‐Prahova, 2006). Child disability is shown to increase uncertainty in a family, which in turn may provide a motivation for parents, more frequently fathers, to leave the union (Cohen & Petrescu‐Prahova, 2006).

Single parents of children with disabilities face unique challenges in raising their children that lie at the intersection of single parenthood and caring for a child with a disability. Single parents and their families are particularly vulnerable to poverty, especially when compared to two-parent families (Brady et al., 2017; McLanahan, 2009), as are parents of children with disability (as highlighted above), leaving single parents of children with disability in a position of particular risk for poverty (Blackburn et al., 2010). Despite their willingness to work (Kim et al., 2023), single parents of children with disabilities have major problems balancing paid work and intense childcare responsibilities, especially in countries where parental leave and public childcare are limited (Kim et al., 2023; Vinck & Van Lancker, 2020). Lack of accessible and specialized childcare, a problem for all parents of children with disability (Porterfield, 2001; Powers, 2001; Reichman et al., 2008), is potentially even more consequential for single parents who depend on external childcare to be able to access and maintain employment (Kim et al., 2023; Porterfield, 2001). Even when they work, single parents of children with disability are more likely to be in low paying (Schormans & Brown, 2004), inflexible or part-time jobs (Porterfield, 2001), because of their lower education (Vinck & Van Lancker, 2020).

Single mothers of children with disability experience higher stress levels (Floyd & Gallagher, 1997) and higher depression levels (Levine, 2009; Olsson & Hwang, 2001) compared to partnered parents of children with disability. They also rely more on support services compared to two-parent families (Floyd & Gallagher, 1997; Levine, 2009), potentially due to the lack of support from a partner, which is instrumental in mediating stress in these families (Kim et al., 2023).

Child disability imposes constrains on the economic and time resources of grandparents, who report negative emotions (Katz & Kessel, 2002) and having to make personal sacrifices to support their grandchildren with disabilities (Hillman et al., 2016). This can reduce the incentives to be an involved grandparent and the support they offer, as compared to grandparents of children without disabilities. These grandparents may also suffer from courtesy stigma, that is the stigma that also affects those who are closely associated to individuals with disability, such as members of the family, who may feel teased, abused, or blamed (Ali et al., 2012; Birenbaum, 1970; Larson & Corrigan, 2008). They may then develop negative emotions, thereby withdrawing from their role as grandparents. It is finally unclear how singlehood is related to support from grandparents, but some studies find that single-parents receive more help from grandparents than married parents (Cooney, 2021).

Based on previous literature and investigations, we formulate a series of expectations, summarised in Table 1.

**Table 1:** Expectations regarding the four-way comparison

Parent group	Employment rate	Poverty rate	Child maintenance receipt	Mental health	Physical health	Social support
Partnered parents, no disabled child (P, reference group)	Highest	Lowest	Highest (if applicable)	Best	Best	Lowest
Partnered parents, disabled child (PD)	Lower than P	Higher than P	Similar to P (if applicable)	Worse than P	Worse than P	Lower than SP but higher than P
Single parents, no disabled child (SP)	Lower than P and PD, higher than SPD	Higher than P and PD, lower than SPD	Less than P and PD, higher than SPD	Worse than P and PD	Worse than PD, better than SPD	Highest
Single parents, disabled child (SPD)	Lowest	Highest	Lowest	Lowest, similar to SP	Lowest	Higher than P and PD but lower than SP

Despite the overlapping disadvantages that leave single parents of children with disability, and their families, in precarious situations, both economically, and in terms of health, there has been little research acknowledging the intersectionality of these issues (for an exception that investigates labour market outcomes see Vinck & Van Lancker, 2020). This paper proposes an investigation of the well-being of single parents with children having a disability by considering the overlapping and intersecting consequences of gender, singlehood, and caregiving a child with a disability.

### Intersectionality

Intersectionality is “a tool that combines separate understandings of various axes of stratification” (Shifrer & Frederick, 2019, p. 1). As it relates to disability, some studies have tried to understand how disability stands at the intersection of poverty, state violence, and dangerous workplaces (Jenkins, 1991; Shifrer & Frederick, 2019) to marginalize people with disabilities. Some studies argue that disability is a stratifying factor, independent of social class, that is often socially constructed (Jenkins, 1991). However, little research has been done in the field (Chatzitheochari et al., 2022), and disability studies often ignore intersecting issues of gender, race/ethnicity, and potential sources of social disadvantages that interact with disability beyond social class.

Even more rare is the investigation of the intersectionality of single parenthood and parenting a child with disabilities, even though some studies have shown that parents of children with disabilities are often single, low-educated, and more likely to have a disability themselves (Vinck, 2022), all characteristics that create disadvantage in social and economic life beyond those associated with having a child with a disability (Vinck & Van Lancker, 2020). Moreover, because in many countries caring for a child with disability is considered women’s responsibility and because fathers show on average lower commitment to caring for children, children with disability are more likely to live with their mothers when the parents are separated, a situation often reinforced by service providers (Cohen & Petrescu‐Prahova, 2006). As a result, single parents of children with disabilities have to navigate gender-related disadvantages as well as singlehood and parenting a child with a disability. In this paper, we attempt to understand the well-being of single parents of children with disability through an intersectional lens that considers the overlap between caring for a child with a disability, singlehood, and gender.

## Data and Methods

This paper relies on data from the UK Millennium Cohort Study, a panel study of 18,818 children born between 2000 and 2001 (response rate: 72%). The panel study follows children from the age of 9 months to 17 years old, for a total of 8 sweeps fielded in 2001, 2003, 2006, 2008, 2012, 2015, 2018, and 2023. We use information from the first 6 sweeps of data collection that include detailed information about parents’ well-being. We exclude the last two sweeps because they are centred on the sampled children and do not include important information about parental well-being. This dataset is very suitable for the aims of the present study because it interviews parents of reference children and compiles a vast amount of information about the characteristics of the child, the household, and the parents, allowing us to investigate the consequences of child disability on parental well-being. Similarly, the sample under study is representative of children born at the beginning of the twenty-first century (birth cohorts 2000 and 2001) in the UK, making this a population-level investigation of disability.

### Family Structure Measurement

To measure single parenthood, we rely on the most common strategy in the literature, which identifies single parents as an adult living with their underaged children but no partner or spouse (Nieuwenhuis & Maldonado, 2018). This measure allows us to exclude cohabitating parents from the definition of single parenthood, as in the UK a cohabitating household is similar to a married household (Mostafa et al., 2018, p. 908). We are also able to include in our sample those single parents who live in doubled-up households (households that include other members besides parents and children, usually referring to multi-generational households) (Harvey, 2020; Harvey & Dunifon, 2023), an important category of single parents who are usually excluded from single parenthood estimations (Moullin & Harkness, 2021). Given that doubled-up single parents are different in many respects, in Appendix 1 we replicate our analysis on a sample of parents who live alone with their children, excluding 9.5% (7852 person-year observations) of households from the main analysis. Our sample of single parents is comprised of all individuals who comply with this definition for at least one sweep. This measure of single-parenthood is not sensitive to custody arrangements, and thus, parents who share custody (equally or not) are included in our definition of single-parenthood, following examples in the literature (Nieuwenhuis & Maldonado, 2018; Zagel, 2023). In Appendix 2, we engage in an additional robustness check by including in the analysis a continuous measure of single parenthood that takes advantage of the longitudinal character of the dataset and computes the proportion of time spent in a single parent family over the 6 sweeps of data collection. This allows us to explore changes in family structure over time, but due to small sample sizes of single parents of children with disabilities, we do not use this strategy in the main analysis. Additionally, we replicate our analysis using a more restrictive design: we only consider parents who comply with this definition in all the years in which they are observed. Our results are robust to either specification of single parenthood.

### Disability measurement

Disability is defined as a condition that limits the ability of the child to undertake everyday activities, based on the Global Activity Limitation Indicator. In this paper, we rely on parental disclosures of a child’s longstanding illnesses. If the parent declared that the illness limits the daily activity of a child to some degree, and this limitation is present in at least two sweeps in the data, we classify this child as having a disability. We choose two years to exclude from our sample children who might have a temporary limitation due to an injury or disease with quick recovery time (for example, broken limbs). In Appendix 3, we replicate the analysis by defining child disability in a less stringent way: a child with a disability is any child whose parents report a limitation in at least one sweep of data collection. A stricter definition for disability, for example someone who reports limitation in all sweeps of data collection, is not possible due to small sample sizes deriving from attrition. Even though this measurement relies on subjective parental perceptions, it has the advantage of being broader than other measurements of disability, which often rely on benefit take-up or medical diagnoses. These measurements often underestimate the prevalence of disability and introduce important selection biases.

### Outcomes of interest

We measure three dimensions of well-being: health, economic, and social well-being. First, to assess the mental health of single parents, we use two different questions (Santor et al., 2006), one that asks respondents how often they felt depressed on a scale from 1 “all of the time” to 5 “none of the time” during the previous year, and one that inquires if the parent is receiving treatment for depression or anxiety (0 “*no*”, 1”*yes*”). We rescale the first variable so that higher score means people reported that they felt depressed more often (with 0 “*none of the time*” as a reference category). These measures cover both subjective and objective measure of mental health and have the potential to uncover differences in access to medical treatment (Suchman et al., 1958). We also use a well-established subjective measure of general health (Manor et al., 2001) of the parents, based on their responses on a question that asks respondents to rate their health from “*poor*” to “*excellent*” on a 5-point scale.

Second, to assess economic well-being, we use parental labour market participation, measured as the non-employment rate of parents, as well as poverty rate. Our measure excludes from employment any activity other than paid, formal employment. The non-employment rate is computed by tolling up the total number of years a person is not employed and dividing it by the number of years they are observed in the sample. We measure poverty using the OECD threshold that classifies a family as poor if their annual equivalized income is lower than 60% of the median income of the UK population in that year. The UK Millennium Cohort Study has an in-built poverty measure that complies with this definition, and due to incomplete and unharmonized information on family income we rely on the variable already present in the data set to measure poverty. Additionally, we compare the receipt of child maintenance payments received by single parents of children with and without disability; to understand to what extent the absent parent is involved in the financial well-being of their children. The data about child support is collected by asking parents “Does absent parent contribute to maintenance?” with 3 possible answers: “*Yes, regular payments*”, “*Yes, irregular payments*”, “*No payments*”. We recode this variable into a binary one to identify those parents who receive any child support payments and those who do not. Receiving child support is also a good, albeit imperfect, indication of the relationship quality between the non-residential parent, the child, and the residential parent.

Lastly, to measure social support from grandparents, we rely on a common strategy in the literature that uses frequency of contact as a proxy for social support (Becker et al., 2019; Kana’iaupuni et al., 2005; Stansfeld & Marmot, 1992; Voorpostel & Van Der Lippe, 2007). We make use of two sets of variables. For the first two sweeps of data collections, the parents were asked how often they had contact with their mother and father (i.e. the grandparents of the reference child). We collapse the two variables together, to get the maximum times parents had contact with either their mother or father. For sweeps 3, 5, and 6, the survey asked how often the reference child saw their grandparents. We then combine these variables to get a social support variable that measure the contact between the nuclear family and the extended family (i.e. grandparents), on a discrete 6-point scale ranging from 1 “*every day*” to 6 “*never*”.

### Sample selection

We pool data from the first 6 sweeps of the UK Millennium Cohort Study because from 2018 onwards the children followed by the survey report their disability status themselves, and the questions asked to their parents are very limited. To keep the measurement of disability constant across the window of observation, and due to our focus on parents, we choose to exclude data from sweeps 7 and 8 which interview children rather than parents. Our initial sample is formed of 82,588 observations from a total of 18,669 unique individuals. A parent must be present in at least two sweeps of data collection to be included in the sample. Some parents have more than one child in the sample in one year, which means they will appear in the dataset more than once in each year. Because our analysis is at parent-level, we must reduce the number of observations for each parent to one. In these cases, we keep the observations for the first reference child if no children or all children have a disability, and for the child with a disability when only one child has a disability.^4^ After identifying the status of single parenthood and eliminating from our sample of single parents those whose household is comprised of one person, we are left with a total sample of 18,481 observations from 4,436 single parents. Of these, 304 parents have children with disabilities, for a total of 1,588 observations over time. An overwhelming majority of the sample (99%) is female. In Appendix 4 we present results from panel fixed-effects regressions that explore changes over time in well-being.

### Comparison Groups

Our results are presented comparatively across four family types: single parents of children with no disabilities (SP, n = 16,893), single parents of children with disabilities (SPD, n = 1,588), the reference category of partnered parents of children with no disabilities (P, n = 60,588), and partnered parents of children with disabilities (PD, n = 3,519). The main focus of the paper are single parents of children with disabilities but the multiple comparisons allow us to put our results into perspective and better understand different sources of disadvantage.

### Analytical Approach

We engage in a descriptive analysis of the differences between parents of children with and without disability, both single and partnered. We consider caring for a child with a disability as a source of heterogeneity in single parenthood and investigate demographic differences between these two groups of single parents, while also relating them to demographic characteristics of two-parent families. We present descriptive results regarding the age, education, parents’ disability, family size, participation in formal childcare, and residential instability. Next, we show results from OLS regression analyses that investigate the association between family type and our outcomes of interest, i.e., different dimensions of well-being, following the general model in Eq. 1. ${Outcome}_{i}$ is any of the outcomes described above for a parent $i$. ${\beta }_{1}$ is the association between ${Outcome}_{i}$ and the $FamilyType$, controlling for ${X}_{i}$ which is a vector of variables that includes year-fixed effects, parents’ education, parents’ age, parents’ disability status, and the number of siblings in the household.

1 $${Outcome}_{i}={\beta }_{1}*FamilyType+{X}_{i}+\varepsilon $$

Because child disability is quite rare in our sample, using person-level data would substantially reduce sample sizes. We therefore use person-year observations. This increases statistical power and allows disability status and parental well-being to vary over time. Standard errors are clustered at the parent level to address non-independence of observations. Similarly, we only present panel-fixed effects models in Appendix 4 because small yearly sample size for (single) parents of children with disabilities makes across time-comparisons uncertain. We are also concerned with the higher attrition rate of parents of children with disabilities and parents with low socio-economic status, which may cause selection bias.

Lastly, we take advantage of the detailed information provided in the survey to investigate common barriers to work for single parents in our sample, as well as accommodations in the workplace that allow single parents to access and maintain a job.

## Results

We start by exploring demographic differences among our four groups of parents. We then move on to understanding differences in (mental) health, followed by an analysis of economic differences and social support. Lastly, we explore mechanisms that could explain differences in non-employment rate.

### Demographic Characteristics

Single parents are often considered a homogenous group; however, their most common characteristic is their heterogeneity (Weinraub & Kaufman, 2019). In this section, we present child disability as a source that introduces heterogeneity in single parenthood, potentially adding a further source of disadvantage, and explore the demographic differences between single parents of children with no disability and single parents of children with a disability. Table 2 offers a summary of this section and presents averages across parent groups.

**Table 2:** Demographic characteristics of parents

Variable	SP	SPD	P	PD	
Age (in sweep 1)	25.9 (6.3)	26.3 (6.2)	30.1 (5.5)	30.3 (5.4)	
Education (% with higher education)	48.7	40.5	60.8	55.5	
Parents’ own disability (%)	17.8	32.7	11.5	28.4	
Number of siblings (excluding the reference child)	1.3 (1.3)	1.3 (1.2)	1.3 (1.1)	1.5 (1.5)	
Hours (per week) spent in childcare facility	2.1 (7.9)	1.6 (6.9)	2.7 (8.7)	2.3 (8.1)	
Number of residence changes	1.6 (0.8)	1.8 (0.9)	1.4 (0.6)	1.5 (0.7)	

Authors’ own calculations based on UKMCS. Standard deviations in parenthesis. Education refers to the % of parents with a higher education degree. A parent with a disability is defined as someone who reports a limitation in daily activities in any sweep; the percentage is polled over all observations across years

Single parents are similar in their average age, education, and number of children regardless of the disability status of their children. Compared to partnered parents, single parents are younger, less educated, and have changed their residence more often. Children who do not have a disability spend more hours per week in formal childcare facilities, such as nurseries, daycare, or preschool, than children with a disability both in single-parent families and in two-parent families. All families seem to have a similar number of children regardless of their structure and child disability status. Parents of children with disability more often have some form of disability themselves, which is likely also due to selection mechanisms according to which frail health conditions may be inherited from parents. However, it is interesting to observe that this proportion is especially high for single parents, indicating the relevance of the intersectionality between child disability and single parenthood.

### Well-Being Outcomes

In this section, we explore how sociodemographic differences are reflected in health and economic outcomes by comparing our four family types.

**Fig. 1:**
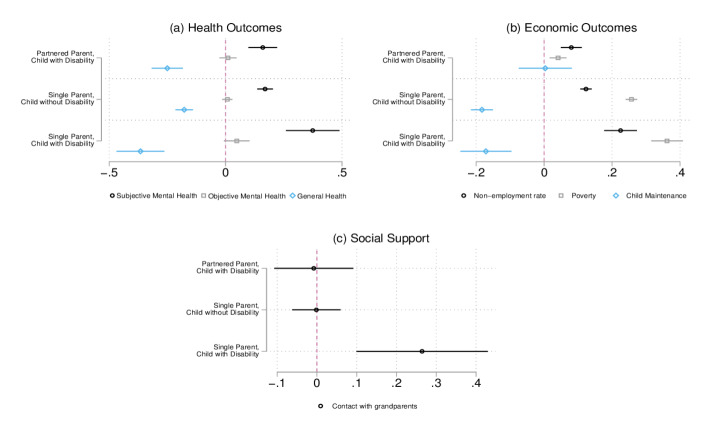
Associations Between Well-Being and Family Type. Authors’ own calculations based on UKMCS. The X-axis represents the linear coefficient interpreted as the association between each family type (from the Y-axis) and the outcome of interest. The regression models control for year-fixed effects, parents’ education, age, and disability status, and the number of siblings in the household

We ran separate regression analyses for each outcome variable, controlling for year-fixed effects, parents’ education, parents’ age, parents’ disability status, and the number of siblings in the household. We present the results in Fig. 1. Partnered parents of healthy children are the reference category for all results.

Panel (a) in Fig. 1 presents all results for health outcomes. Across health outcomes, parents of children with disability report lower well-being than parents without children with disabilities. Partnered parents of children with no disability have the highest levels of general and mental health, confirming our expectations. Partnered parents of children with disability report feelings of depression (subjective mental health) 16 percentage points (p.p.) more often compared to partnered parents of healthy children and to a similar degree as single parents of children with no disability.

Single parents of children with a disability have the worse subjective mental health outcomes, with a probability of reporting to feel depressed which is almost 40 p.p. higher compared to the one of partnered parents of healthy children and 20 p.p. higher than single parents of children with no disability. This indicates the intersection between being single and having a child with a disability which increases feelings of depression to a higher degree than each of these dimensions alone.

The likelihood of receiving treatment for anxiety or depression is small across all groups, with no statistically significant differences. The results for objective mental health confirm the trends in subjective well-being, with single parents of children with no disabilities having similar levels as partnered parents of children with disabilities, and lower than single parents of children with disabilities. Once again, the intersection between singlehood and having a child with a disability could explain these results.

Regarding general health, Fig. 1, Panel (a) reveals different results. Single parents of children with no disability have the second highest (i.e., best) rating on subjective health, with 18 percentage points less than partnered parents of children with no disabilities. Parents of children with disability report worse self-rated health: compared to the reference group of partnered parents of healthy children, it is 25 percentage points lower for partnered parents and 37 percentage points for single parents of children with a disability. This might not be surprising as more parents of children with disability have a disability themselves (see Table 2).

In Fig. 1, Panel (b), we present the regression results for associations between family types and economic well-being outcomes. As opposed to health outcomes, across all economic outcomes, single parents report lower well-being. The non-employment rate of partnered parents of children with disabilities is 8 p.p. higher as compared to partnered parents of healthy children. This is not surprising, as most of our sample is made up of mothers, and, as previous research has shown, coupled mothers of children with disabilities decrease their participation in the labour market to take care of their children (Jenkins, 1991; Powers, 2001; Reichman et al., 2008). Single parents have higher levels of non-employment compared to partnered parents, especially if their child has a disability: single parents of children with disabilities have a non-employment rate higher with 23 p.p. relative to the references group, and 12 p.p. higher compared to single parents of children without a disability. This is also reflected in the highest probability of living in poverty for single parents of children with disabilities, confirming our expectations. Across the six sweeps, these families have a probability of living in poverty that is 36 p.p. higher compared to partnered parents of healthy children. Instead, single parents of healthy children have a likelihood of being in poverty that is 26 p.p. higher than the reference group. Even though partnered parents of children with disabilities have low employment rates, they also have relatively low poverty rates, only 4 percentage points higher compared to partnered parents of children with no disabilities.

Single parents are less likely to receive child maintenance compared to partnered parents. Regardless of the disability status of their children, single parents received child maintenance 18 p.p. less than partnered parents. This indicates not only a lack of financial support from the non-residential parents but likely also their low involvement in the life of the child.

For economic outcomes, partnership status seems to be more important than the disability status of children, especially regarding the likelihood to receive child maintenance. However, single-parents of children with disability still experience somewhat lower economic well-being, indicating some intersectionality.

Lastly, Panel (c) in Fig. 1 presents the association between family type and the frequency of contact with extended family, a proxy measure of social support from grandparents. Compared to partnered parents (with or without disabled children) and single parents of children with no disability, single parents of children with disability receive more social support from grandparents. The increase in social support if about 30 p.p. indicating that it is the intersection of marital status of the parent with the disability status of the child that substantially increases social support, not supporting our expectations. This is confirmed by the small and insignificant difference in contact between partnered parents of children with disabilities, partnered parents of children without disabilities and single parents of children without disability.

Although group differences are robust to different measures of family structure and disability, our sensitivity analyses highlight three important additional points. In Appendix 1, we exclude doubled-up single parents from our definition of singlehood. This exclusion slightly reduces the association between single-parenthood and well-being, indicating that doubling-up could be a strategy for dealing with the negative consequences of singlehood and of raising children with disabilities. Similarly, in Appendix 2, we highlight that the length of singlehood matters for well-being outcomes, with lower levels of well-being among parents who are single for the entire observation period as compared to those who are single for only parts of the observation period. Appendix 3 replicates the main analysis but relaxes the restriction on the number of years a child needs to report a limitation for them to be categorized as having a disability to only one year (instead of 2 or more). These results slightly reduce the association between child disability and parental well-being indicating that a more permanent disability status is more detrimental to parental well-being compared to temporary limitations.

### Mechanisms for differences in employment

Parents of children with a disability are less frequently employed compared to parents of children with no disabilities. But are these differences a reflection in preferences or are they results of structural and institutional barriers? Single parents experience important barriers to work, especially due to their double role as main caregivers and breadwinners but single parents of children with a disability are potentially even more challenged by this double role. In this section, we explore the mechanisms behind employment differences between (single) parents of children with and without disabilities.

**Fig. 2:**
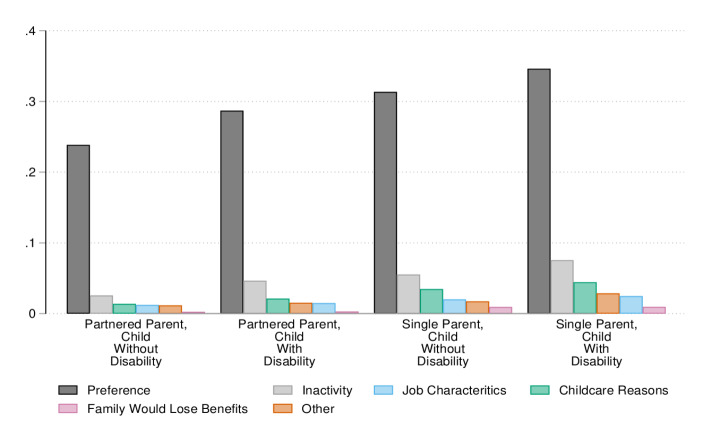
Barriers to Work. Authors’ own calculations based on UKMCS. The percentages are calculated from the total number of people who are unemployed (30% of the total sample). The data is pooled from all 5 sweeps (except sweep 3) in which this variable was collected

This section’s results are based on detailed cross-sweep questions that ask respondents to provide reasons for why they are not employed. We collapse over 30 different possible responses into six main categories. First, preference refers to all individuals who are not employed because they: i) prefer not to, ii) prefer to look after their children themselves or to be at home with the family, or iii) said they are taking care of a child with disability or another family member. The inactive category includes all individuals who are inactive due to educational reasons, such as participating in full-time education or training, including Governmental schemes, have poor (mental) health, are pregnant, or are on parental leave. Job characteristics reasons are all reasons that are related to the job market, such as the inability to find available jobs, jobs in the right hours, jobs that pay enough, or jobs that are in the right area. Next, the “childcare reasons” category convenes those individuals who do not work because they cannot find affordable or suitable childcare. We create a category for individuals who do not work because their family would lose their welfare benefits if they did. Every other reason, such as the fact that their partner disagrees or works unpredictable hours, language barriers and lack of work permits is found under the “other category”. The share of individuals by type of barrier is provided in Fig. 2.

First, regardless of the disability status of their children, the most common reason single parents provide for not being formally employed is that they prefer to take care of their children by themselves. This is also the most common reason among partnered parents but in lower degrees. Parents of children with a disability prefer not to work more often compared to parents of healthy children, regardless of their family structure. However, almost 35% of single parents of children with disability are out of employment for this reason, compared to 30% of both single parents of children with no disability and partnered parents of children with disability, and 23% of partnered parents of healthy children. These results are based on self-reported reasons for not working, however we acknowledge that preferences are often socially constructed and that many times parents of children with disability do not have viable option for childcare other than doing it themselves (Ellem et al., 2016).

The second most common reason (but with shares lower than 10% for each family type) is inactivity of some form. This is again more often a reason of non-employment among single parents compared to partnered parents, and for parents of children with disability compared to parents of healthy children.

One of the most important institutional barriers to work is being unable to find suitable or affordable childcare which is more common among single parents. This is indicative of their struggle in balancing childcare and work responsibilities and having to leave the labour market to take care of their children. Job characteristics are also more common among single parents, who potentially have stricter job mobility possibilities and require special work hours to accommodate their increased care responsibilities. Job characteristics are only reported by about 1% of partnered parents, as opposed to 2.5% of single parents of children with disabilities. Losing welfare benefits is the least common reason for all family types, indicating that single parents are not relying on welfare as an alternative to work.

## Discussion and Conclusion

This study highlights how the intersection of single parenthood and child disability creates a double challenge with negative consequences on health and economic well-being of parents. We argue that single parents of children with disabilities face unique challenges that have been largely overlooked both in research and by policy makers. Using longitudinal data from the UK Millennium Cohort Study, we descriptively show that single parents raising children with disabilities grapple with mental and physical health struggles, poverty, and limited employment opportunities, exacerbated by the intense caregiving responsibilities that accompany raising a child with a disability.

Our findings show that single parents of children with disabilities have worse mental and general health than both partnered parents having children with disability and single parents with healthy children. The same is found for different dimensions of economic well-being: single parents of children with disability are the least likely to be employed and to receive economic support from the non-resident parent, and the most likely to live in poverty. On the other hand, single parents of children with disabilities receive more social support from their own parents, indicating that the disability and partnership status of parents work together as an incentive of extended family members to help out.

Our descriptive analyses further highlight that the likelihood of being non-employed is shaped primarily by parental preferences (even if these preferences are the result of social norms), and only then by lack of conditions that could favour better work-family reconciliation. Indeed, the main reason why these parents are not employed is because they prefer to take care of their children. This reason for being non-employed is common for all types of families, but especially for single parents who have a child with a disability. This could indicate that caregiving for a child with a disability is strongly linked with social norms, particularly gendered expectations around caregiving, which play a crucial role in shaping employment decisions. Although social norms evolve slowly, structural and institutional barriers driving employment disparities can and should be addressed through social policy. Employment not only provides stable income and reduces poverty (Gornick et al., 2022) but also enhances social interaction, fulfilment, and self-esteem, supporting parents’ mental health (Kim et al., 2023). Given these benefits and parents’ clear desire to work (Kim et al., 2023), in Appendix 5 we show that while all parents rely on flexible work accommodations to reduce work–family conflict, single parents—especially those with children with disabilities— who could benefit from these accommodations the most, have the least access to them.

Overall, our findings clearly shows that it is very much the intersection of the two sources of disadvantage under study, that is single parenthood and child disability, that leads to the lowest well-being for families. The economic well-being and social support of single parents of children with disability is most clearly influenced by the intersection of both statuses. However, some determinants of lower well-being seem to be driven mainly by single parenthood, such as a low child maintenance or the unmet need of more flexible work accommodations (see Appendix 5), others are instead clearly driven by child disability, such as lower perceived mental health. Moreover, our descriptive findings show that parents of children with disability are overall more likely to have a disability themselves, a piece of evidence that suggests a potential selection mechanism. However, being at the intersection with single parenthood associated with the highest probability of having a parental disability, we could speculate that the partnership status could further worsen an already fragile health of these parents.

Our study has several limitations. First, our results are descriptive and as such they cannot and should not be interpreted causally. Children with disabilities are a highly selected group, as are their families and causal identification is particularly difficult to achieve when studying these groups. While we acknowledge this limitation, we also argue that the aim of this study is not to identify the causal links between child disability and parental well-being, but rather to describe the reality, as is experienced by these families. We argue that regardless of the directionality of the relationship, improving the well-being of (single) parents of children with disabilities cannot be achieved without rigorous and accurate descriptions of their vulnerabilities and needs.

Contrary to common ideas that disability triggers poverty, some studies show that poverty during childhood may have detrimental health consequences due to limited access to health services, poor health behaviour, and dangerous living conditions (Fujiura & Yamaki, 2000; Shifrer & Frederick, 2019; Vinck, 2022) challenging the directional link between poverty and disability. This can also be true for the rest of our outcome variables.

Our results probably differ based on sociodemographic characteristics, but due to small sample size of single parents of children with disabilities, as well as many missing information on key variables, such as education, we cannot explore these differences. However, there are reasons to speculate that the mechanisms that explain why some parents do not work could be different based on educational level, with highly educated women potentially more likely to prefer employment over childcare. Further research could explore how these mechanisms vary with educational level and other sociodemographic characteristics.

Although we provide some evidence of longitudinal trends in Appendix 4 and highlight the small changes in our outcome variables over time, due to small sample sizes and few and far apart data collection times, we cannot make inferences about longitudinal trends. Future research could investigate the longitudinal trends in the well-being of parents of children with disabilities.

Despite these limitations, this research highlights the relevance of investigating the spillover effects of child disability on family members, and especially parents. By bridging two previously disconnected areas of study—single parenthood and child disability— it reveals how these intersecting factors contribute to the creations of a double challange for single parents of children with disabilities. Our study advances our understanding of this population by looking at several facets of well-being beyond labour market, that is the focus of much previous research. In doing so, we provide initial evidence of potential structural factors driving the well-being of this population, which in turn suggests implementable institutional changes that can encourage employment among single parents of children with disabilities. Our results underscore the importance of policies promoting flexible work arrangements, accessible childcare, and financial support for single parents of children with a disability are essential in mitigating their unique challenges and improving their well-being.

Lastly, our study is the first one that compares single parents of children with disabilities, to other traditionally and well-studied vulnerable groups, single parents and partnered parents of disabled children and benchmarks these results to those of partnered parents of children without disabilities. By doing this, we highlight the importance of considering the intersectional nature of raising a child with a disability while being a single parent, the unique challenges of these parents, as well as ways to improve their well-being.

## Supplementary Information

Supplementary Material

## Data Availability

The data used in this paper is available from Centre for Longitudinal Studies at 10.5255/UKDA-Series-2000031. The Economic and Social Research Council funds the Centre for Longitudinal Studies Resource Centre (ES/W013142/1) which provides core support for the CLS cohort studies. While the CLS Resource Centre makes these data available, CLS does not bear any responsibility for the analysis or interpretation of these data by researchers.

## Footnotes

1 Evidence on the effect of child disability on union dissolution is mixed (Anchesi et al., 2023; Müller et al., 2022), mainly because of selection bias and differences in disability definition. However, most authors agree that union dissolution is more common among parents of disabled children.

2 Throught this paper, we use the label ‘single parents’ rather than ‘single mothers’ (except when referring to studies that only investigate single mothers), a more common label in previous studies. We do this for two important reasons. First, we argue that talking about single mothers has become improper, as the share of single fathers is increasing. Second, stigmatization of single mothers is common in previous studies and is partly an issue of gender discrimination. Promoting a gender-neutral language is key in the process of destigmatization. However, due to data limitations, our sample is mainly composed of single mothers.

3 Because families typically have more than one child, the number of children is higher than the number of families making the proportion of families with at least one disabled child higher than the proportion of children with disabilities.

4 We do not engage in within-family analysis because the sample size of single parents with disabled children and siblings present in the data is too small for a meaningful quantitative analysis.
