# Supplementary material for: The Double Challenges of Single Parents Raising Children with Disabilities
Source: Eur J Popul. 2026 Feb 17;42(1):9. doi: 10.1007/s10680-026-09767-9 (PMC12982819; doi:10.1007/s10680-026-09767-9)
Supplement: Supplementary file 1 — Supplementary Material [file 10680_2026_9767_MOESM1_ESM.pdf]

Supplementary Material for "The Double Challenges of Single Parents Raising Children with Disabilities"

Burciu Roxana-Diana[2], https://orcid.org/0009-0001-1363-0947

Balbo Nicoletta[1]

[1]Dondena Centre and Department of Social and Political Science, Bocconi University, Milan, Italy

[2]Centre d'Estudis Demogràfics, Barcelona, Spain

Corresponding Author: Burciu Roxana-Diana, rburciu@ced.uab.es

Appendix 1. Excluding doubled-up families

In this appendix, we replicate our main regression analysis by excluding households with additional adults (beyond the householders and their partner) from our sample. Doubled-up households are different in many regards, and these differences might influence the results of our associations. This criterion excludes 9.5% of the households included in the main sample.

Figure A1. Regression Analysis Results

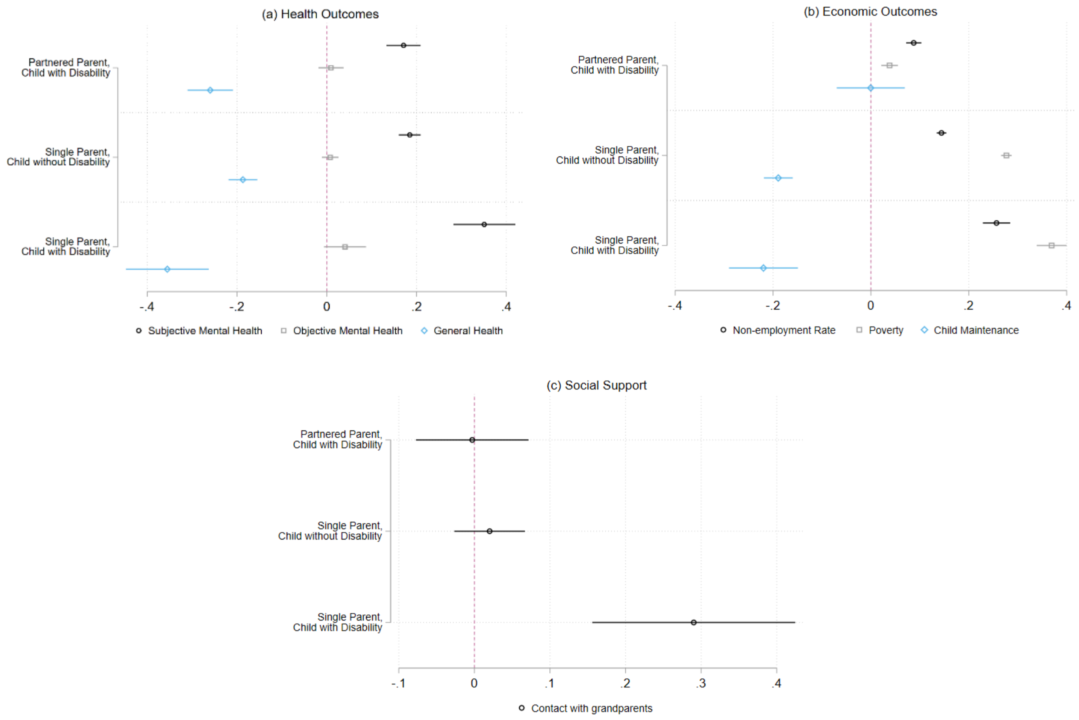

Notes: Authors' own calculations based on UKMCS. The X-axis represents the linear coefficient interpreted as the association between each family type (from the Y-axis) and the outcome of interest. The regression models control for year-fixed effects, parents' education, age, and disability status, and the number of siblings in the household.

Results are mostly similar, with slight increases in the strengths of the associations, especially among single parents of children with disability.

Appendix 2. Alternative measures for single parenthood

First, we provide alternative results for our main analysis that use a continuous measure of single parenthood (as the share of the total number of observed years in which a parent is single). The reference category is partnered parents with children without disabilities, and the coefficients represent those who spend the entire time they are observed as single parents.

Figure A2.1. confirm our main results that both single parenthood and having a child with disability are associated with worse health and economic outcomes compared to partnered parents and parents of children without disability, they indicate an important additional aspect of this relationship: there seems to be no interaction effect between single parenthood and having a child with disability. In other words, these two layers of challenge, although individually reducing well-being among those who experience them, do not reinforce each other.

Figure A2.1. Regressions results with continuous measure of single parenthood

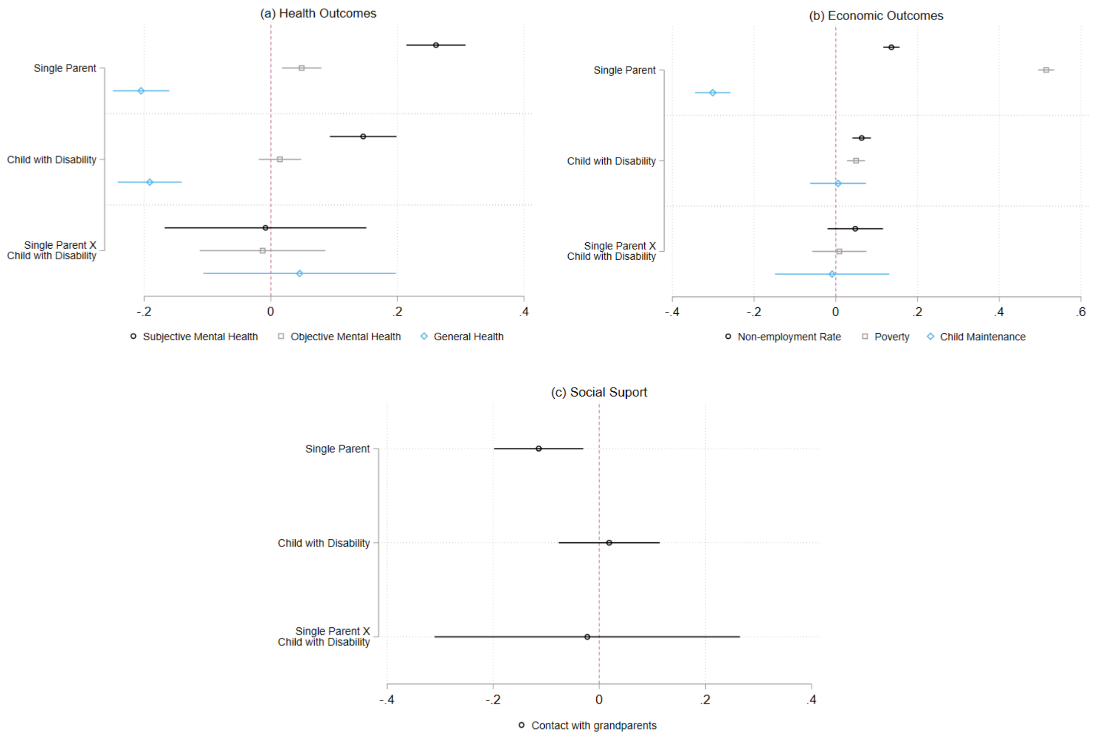

Notes: Authors' own calculations based on UKMCS. The X-axis represents the linear coefficient interpreted as the association between each family type (from the Y-axis) and the outcome of interest. The regression models control for parents' education, age, and disability status, and the number of siblings in the household.

Next, we also present alternative results with a stricter requirement for single parenthood: to be included in the single parent group, an individual must comply with our definition for single parenthood for all the sweeps of data collection. This creates a sample of single parents who are permanently single, and emphasizes their vulnerability compared to those who are temporarily single. Figure A2.2 indicates the increased vulnerability of

permanent parents, for whom the associations are even stronger as compared to those in our main analysis.

Figure A2.2. Regressions results for permanently single parents

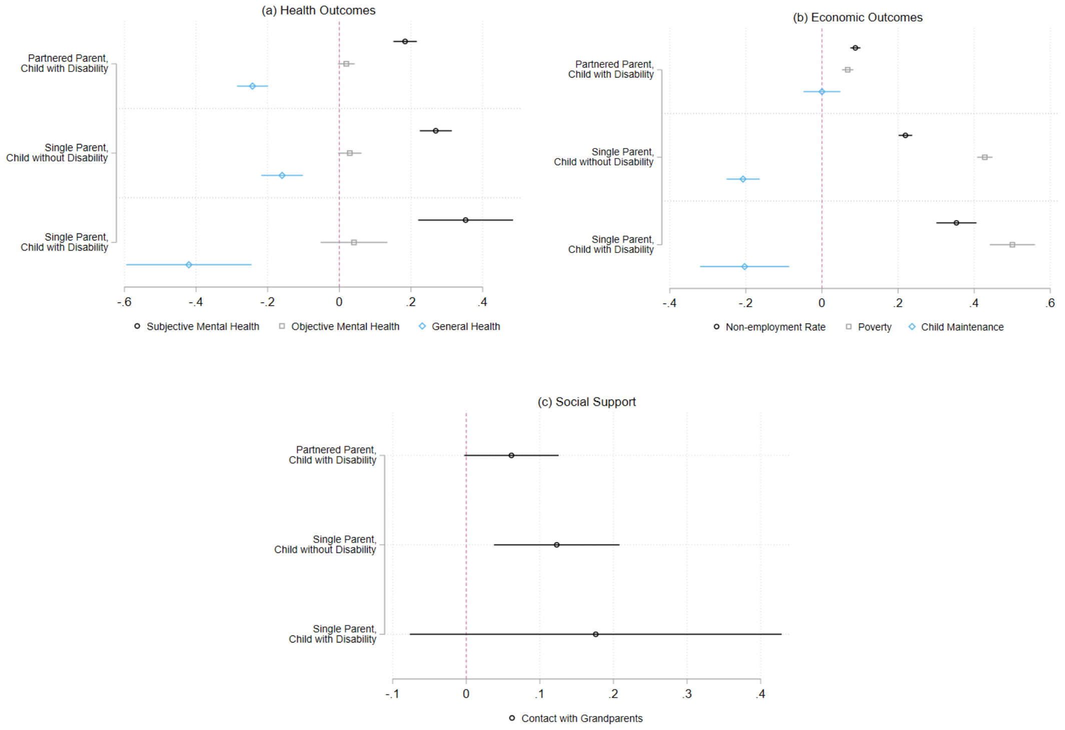

Notes: Authors' own calculations based on UKMCS. The X-axis represents the linear coefficient interpreted as the association between each family type (from the Y-axis) and the outcome of interest. The regression models control for year-fixed effects, parents' education, age, and disability status, and the number of siblings in the household.

Appendix 3. Alternative measure for disability

In our main analysis, for a parent to be included in the group of parents of children with disability, they must report limitations for their children for at least two years of the entire period of data collection. In this appendix, we replicate our regression analysis by including any parent who reports a child's disability in at least one sweep.

The results are similar, but the associations are slightly less strong, indicating that more permanent disability statuses have more negative effects on parents as compared to temporary disability.

Figure A3. Regression results for alternative disability criteria

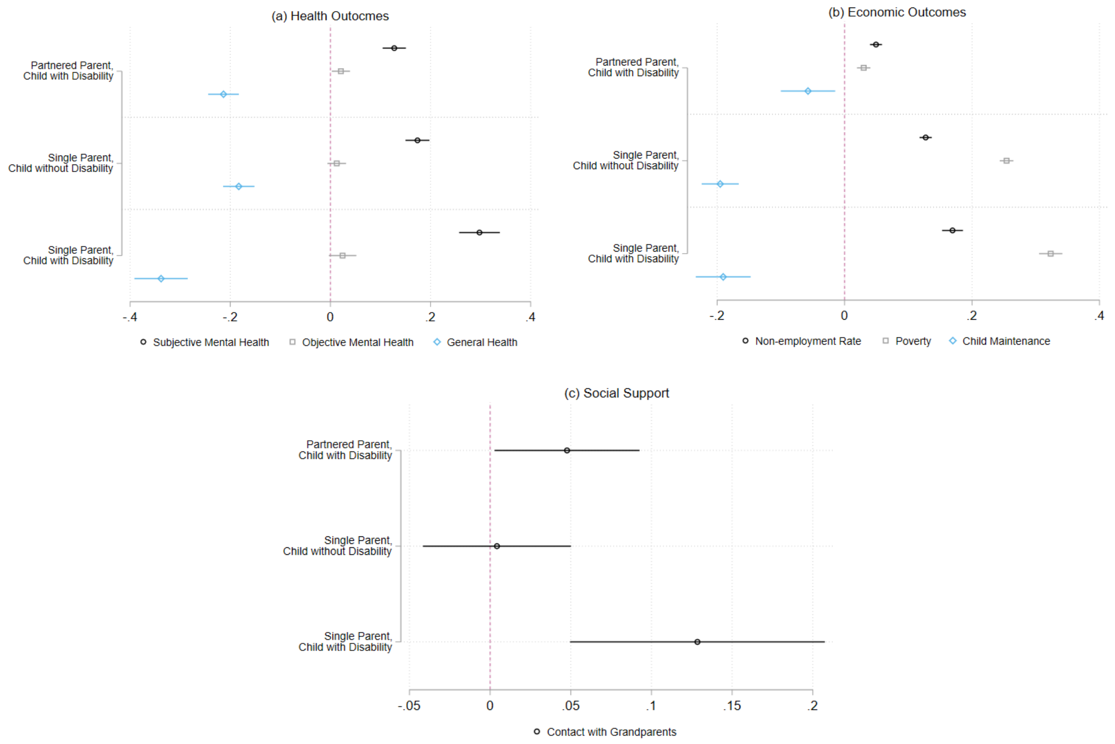

Notes: Authors' own calculations based on UKMCS. The X-axis represents the linear coefficient interpreted as the association between each family type (from the Y-axis) and the outcome of interest. The regression models control for year-fixed effects, parents' education, age, and disability status, and the number of siblings in the household.

Appendix 4. Panel fixed-effects regressions

Figure A4. Over time changes in well-being

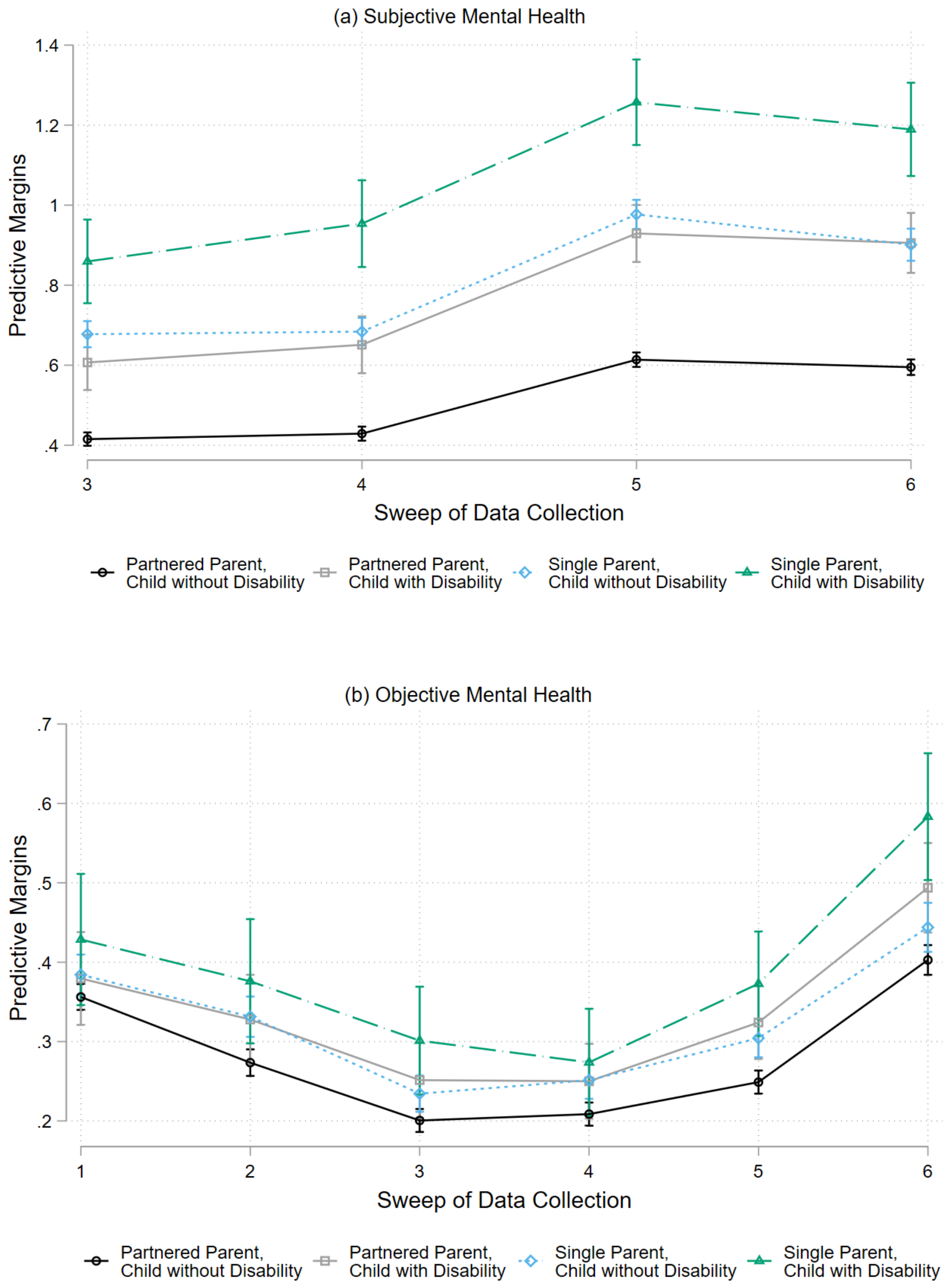

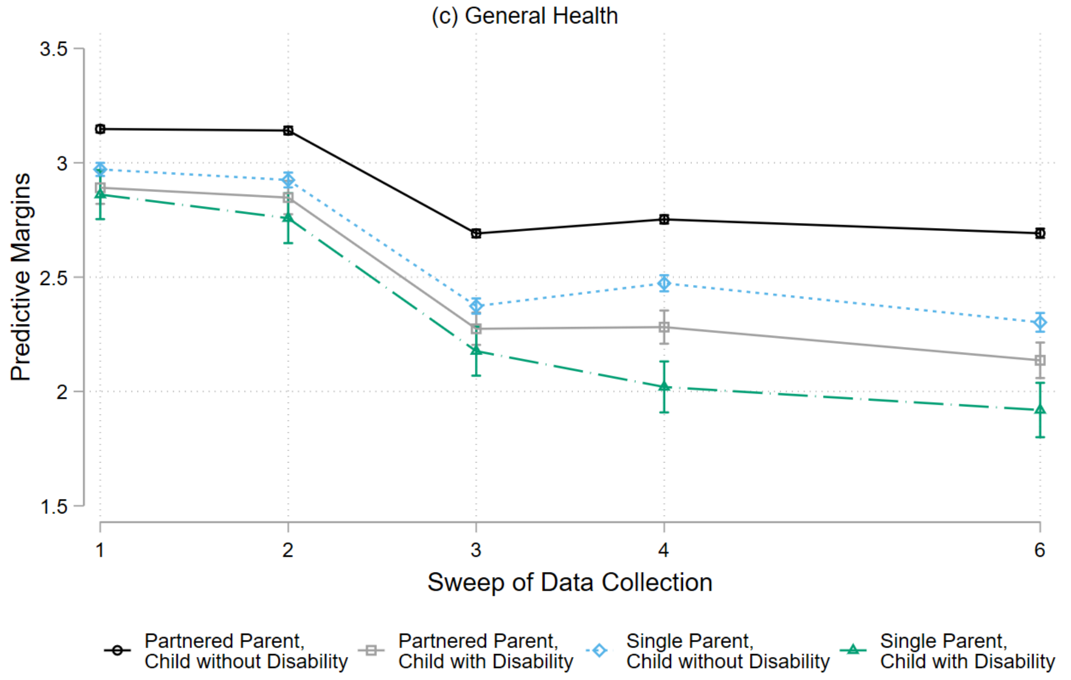
(c) General Health
Predictive Margins
3.5
3
2.5
2
1.5
1
2
3
4
6
Sweep of Data Collection
Partnered Parent, Child without Disability
Partnered Parent, Child with Disability
Single Parent, Child without Disability
Single Parent, Child with Disability

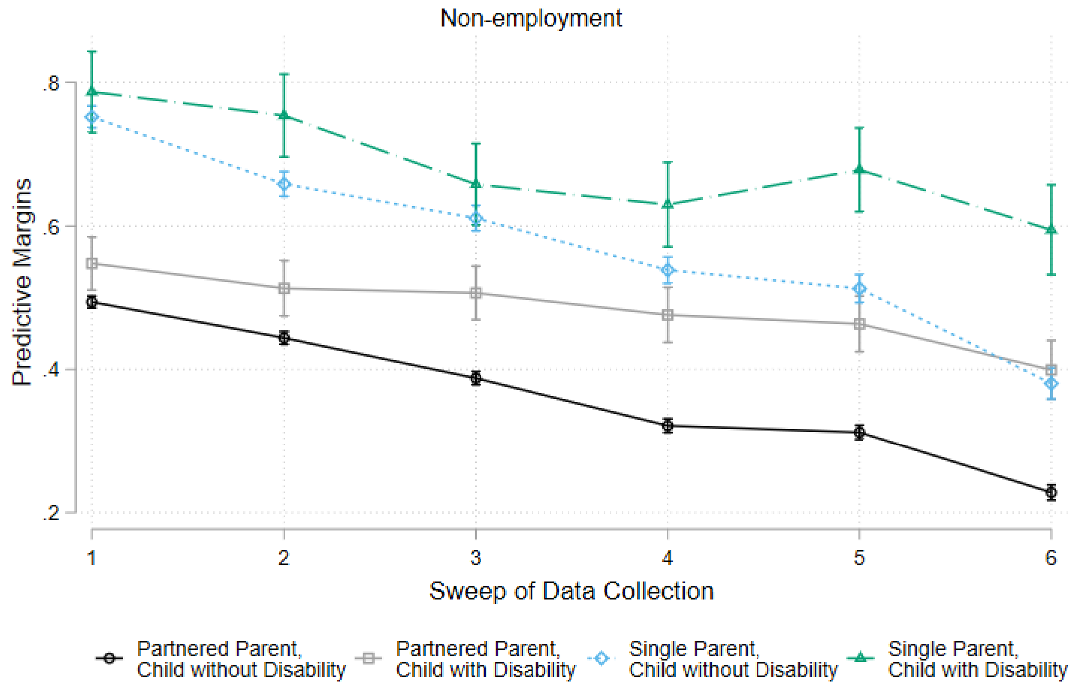
Non-employment
Predictive Margins
.8
.6
.4
.2
1
2
3
4
5
6
Sweep of Data Collection
Partnered Parent, Child without Disability
Partnered Parent, Child with Disability
Single Parent, Child without Disability
Single Parent, Child with Disability

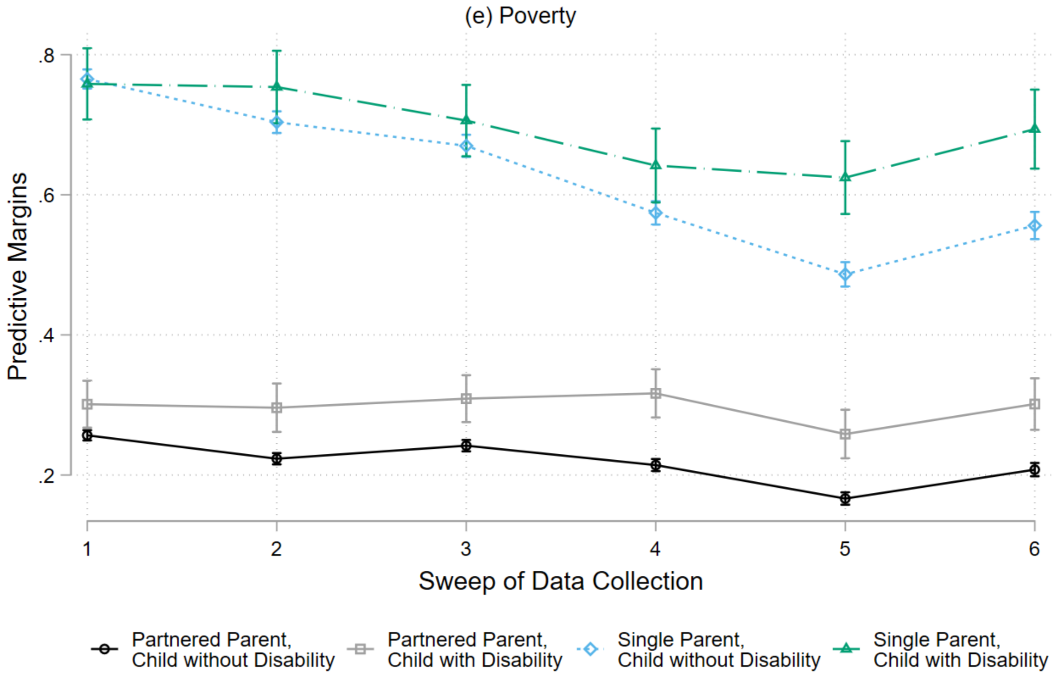

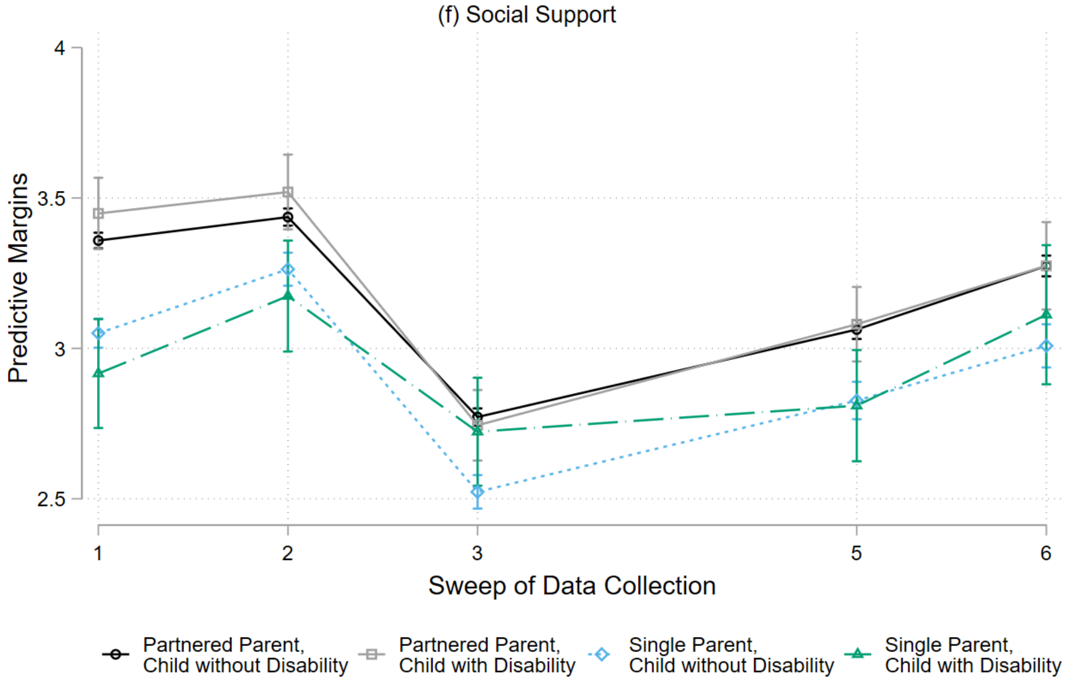

Notes: Authors' own calculations based on UKMCS. Y-axis represents the marginal effects based on panel fixed-effects regression models.

Appendix 5. Improving access to employment for parents of children with disabilities

In this Appendix we explore ways to reduce structural and institutional barriers to work and improve access to employment for parents of children with disabilities through social policy and organisational structure. We do this by exploring the work accommodations used by employed single parents, of which a majority are employed (53%). What work-related characteristics allow these parents to better manage both the responsibilities of childcare and work? Figure A5 provides interesting evidence that can help addressing this question.

The first, and potentially most consequential result, is that single parents use less accommodation in their workplace. This is likely an indication of the lack of access to flexible work arrangements, due to single parents being more often employed in low-income jobs that do not offer non-financial rewards to their employees. Partnered parents of healthy children are most likely to use part-time arrangements (13% do so) but this is less common among all other types of families. In fact, less the 7% of single parents use this accommodation, and only 4.5% of single parents of children with disability use part-time work arrangements. For single parents, and especially single parents of children with disability, the most important work accommodations that allows them to maintain employment is flexible work hours. Single parents also use time off for emergency reasons and other emergency management accommodations, such as personal phones, to balance competing responsibilities of childcare and work. This is also common among partnered parents. Help with childcare, either in the form of payments, or workplace nurseries is important for all types of families, but again, despite their increased need to childcare help, single parents use this less often, potentially due to lack of offer from their employers. Parental leaves are used to about the same extent by all types of parents, indicating their importance in allowing parents to balance family and work. Working from home, on the other hand, is very uncommon among single parents, but more common among partnered parents, again indicating major differences in access to such work accommodations.

Potentially the most important result of Figure A5 is that all parents use and require work accommodations that promote flexibility and help reduce work-family conflicts, regardless of their family structure. Although the need is higher for single parents for such accommodations, they are also the ones who are offered these accommodations least frequently. This is especially true for single parents of children with disabilities.

Figure A5. *Work Accommodations Used by Employed Parents*

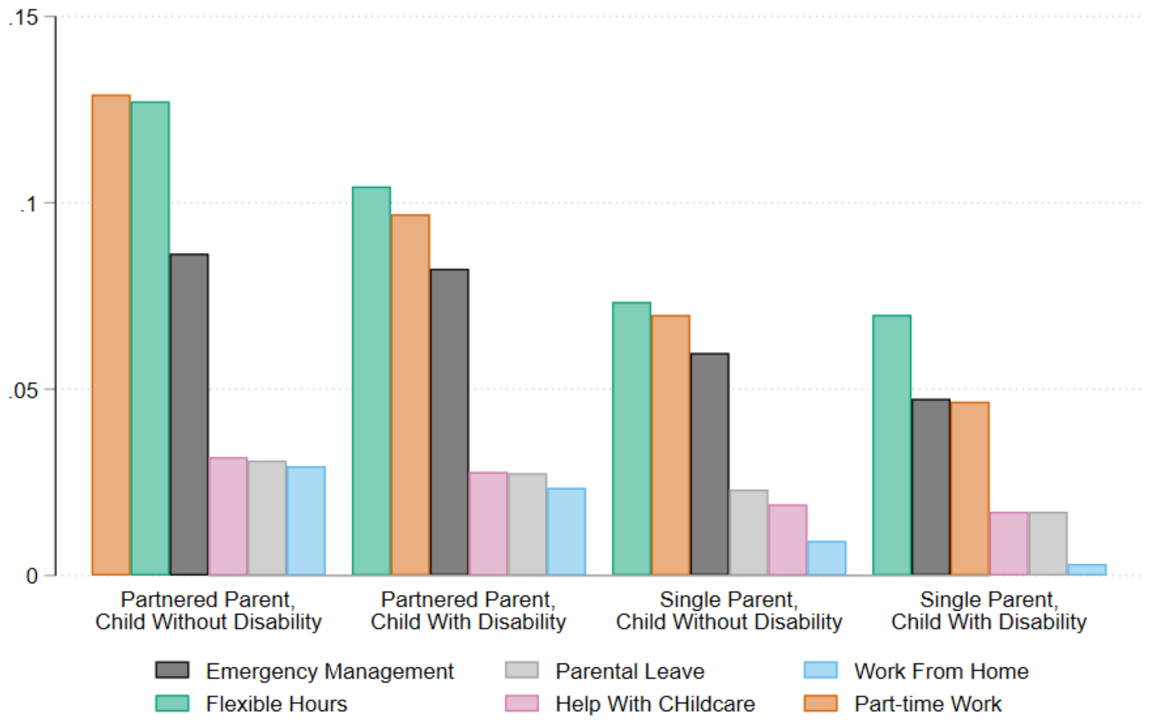

Notes: Authors' own calculations based on UKMCS. The percentages are calculated from the total number of people who are employed (70% of the total sample). The data is pooled from all 5 sweeps (except sweep 6) in which this variable was collected.
